# The research landscape of bipolar disorder in Germany: productive, but underfunded

**DOI:** 10.1186/s40345-024-00344-9

**Published:** 2024-06-15

**Authors:** Cindy Eckart, Andreas Reif

**Affiliations:** 1 Department of Psychiatry, Psychosomatic Medicine and Psychotherapy, Goethe University Frankfurt, University Hospital, Heinrich-Hoffmann-Str. 10, 60528 Frankfurt, Germany; 2 German Society for Bipolar Disorders (DGBS e.V.), Heinrich-Hoffmann-Str. 10, 60528 Frankfurt, Germany; 3 Fraunhofer Institute for Translational Medicine and Pharmacology ITMP, Theodor-Stern-Kai 7, 60596 Frankfurt am Main, Germany

**Keywords:** Bipolar disorder, Germany, Research landscape, Bibliometric analysis

## Abstract

**Background:**

The recurrent mental illness bipolar disorder is a major burden on the healthcare system, which underlines the importance of research into this disease. Germany is one of the most productive countries in this research activity. This bibliometric analysis aims to outline the social and conceptual structure of the German research landscape on bipolar disorder over the last decade. Furthermore, we provide a short overview over current public funding.

**Results:**

Concerning the social structure, most of the German publications were collaboration projects, both with a national but also international orientation, in the latter case predominantly with countries of the global North. Analysis of the conceptual structure of German research activity identified psychiatric genetics, early recognition of bipolar disorder, neuroimaging, and pharmacological interventions as important topics within the field. In the context of a survey, only few publicly funded research projects were reported, many of which did not exclusively investigate bipolar disorder but followed a transdiagnostic approach.

**Conclusions:**

Our bibliometric analysis revealed internationally well-networked German research activities on bipolar disorder. In stark contrast to its high prevalence and correspondingly high financial burden to the healthcare system, current grant support for research on this illness is strikingly low, particularly concerning the development of novel treatments.

**Supplementary Information:**

The online version contains supplementary material available at 10.1186/s40345-024-00344-9.

## Introduction

Bipolar disorder is a mainly progressive mental illness in which the patient’s mood and activity levels are characterized by severe, episodic fluctuations. With a lifetime prevalence about 1–2% worldwide (Merikangas et al. 2011; Rowland and Marwaha 2018) and a high subjective impairment of the affected persons themselves as well as their families and caregivers (Ogilvie et al. 2005; Pompili et al. 2014), it represents a high burden for the health care system. That bipolar disorder is also associated with high premature mortality (Crump et al. 2013; Hayes et al. 2015), partly due to an exceptionally high suicide rate (Dome et al. 2019; Gonda et al. 2012), underscores its medical urgency. This importance has resulted in a vast number of academic publications on bipolar disorder, many of which were also produced under German participation.

The emergence of scientific databases such as Web of Science (WOS), facilitates the access to large volumes of scientific publications. Bibliometric analysis is a comprehensive approach – including quantitative and qualitative methods – to summarize large volumes of research publications and to depict the state-of-the-art of a specific research field by providing insights into characteristics of its particular research outputs. This allows to evaluate the productivity and popularity of individual authors, to visualize connections amongst institutions or authors by collaboration networks (social structure), and to identify important themes and key concepts as well as their evolution into future trends (intellectual / conceptual structure). Such a representation of the scientific landscape of a particular country allows not only to highlight the significance of this country for the research of a particular topic but also to compare it with other countries.

So far, very few bibliometric analyses specifically targeted research on bipolar disorder. Grover and colleagues described the contribution of the Indian research community on the field (Grover et al. 2021). However, this work was mainly concentrated on the general impact of Indian research activities and did not provide a description of the conceptual structure of this community. In somewhat earlier reports, a relative dominance of pharmacological studies was reported (López-Muñoz et al. 2006) and later complemented by the emerging topic of psychiatric genetics (Vogelzang et al. 2012). However, the last decade was not covered in any of these research papers, even though a high number of world-wide publications continue to be published on the topic of psychiatric genetics (Zakaria et al. 2023).

Germany was ranked third (after the USA and the UK) amongst the countries with the most publications on bipolar research (Vogelzang et al. 2012). Accordingly, conceptual analysis of the German research landscape can provide important insights into current trends within the whole field. The main aim of this bibliometric analysis was thus to identify the conceptual structure of current (i.e., published within the last decade) research projects under participation of German scientists and to identify ‘hot’ topics that have particularly shaped the field. Furthermore, we aimed to describe the social network structure of this scientific community and to measure German research performance and relevance in terms of institutions, authors, and journals. To complement this bibliometric approach and to prove substantiated recommendations to funding agencies, we aimed to provide an overview of ongoing publicly funded research projects in Germany. This was also done to get an estimate on the share of research support targeting bipolar disorder and whether it can be deemed adequate.

## Methods

### Data acquisition and search strategy (bibliometric analysis)

Data was retrieved from the Clarivate Web of Science Core Collection (WOSCC; http://www.webofknowlegde.de). Literature search targeted research in the field of bipolar disorder that has been published by authors affiliated in Germany on November 22, 2022, by searching for the terms “bipolar disorder” [topic] AND “Germany” [address] AND 2012-01-01 to 2022-11-22 [date range]. 2,060 results were retrieved by this search and then refined by excluding Meeting Abstracts, Editorial Material, Book Chapters, Corrections and Letters. The remaining 1,763 articles (regular articles, reviews, and early access) were exported as plain.txt file (including full record and cited references) for further analysis with the free R package Bibliometrix (Aria and Cuccurullo 2017) and VOSViewer, a software tool for visualization of bibliometric networks. The data underlying the following analysis (i.e., the full list of results) can be found in the supplement. (*For comparative classification*: An equivalent search without limitation to “Germany” on July 28, 2023 yielded 27,940 results, i.e., authors affiliated to Germany contributed to 6.31% of research published on bipolar disorder world-wide.)

### Data analysis (bibliometric analyses)

Bibliometric analyses were performed with R (version 4.0.5), the bibliometric software packages bibliometrix (version 4.0.1) and VOSViewer (version 1.6.19). Initially, descriptive information for the included article database were retrieved, including annual growth rate of publications and country contribution. We captured the social structure of the German scientific landscape of the last decade by describing the most important institutions, their collaboration network, the most productive authors, their collaboration network, and the most important sources in which their work was published. As recommended by Aria and Cuccurullo (Aria and Cuccurullo 2017), productivity of authors were judged by total number (N) of publications, their frequency of being cited by other authors (TC) and – as a combination of both – their h-index regarding the present publications on bipolar disorder within the last decade (i.e., the h-index is based on the set of a scientist’s most cited papers and the number of citations that they have received in other publications). The conceptual structure of the field was described by evaluating the co-word analysis as well as the thematic map of the most frequently used author key words. Co-word analysis is a mere measure of similarity, in which a semantic map of the research field is constructed by quantifying the co-occurences of frequent keywords. The thematic map, on the other hand, uses sub-group analysis to identify specific themes that are prominent in the field and to group them according to their development (i.e., cohesiveness or sophistication) and their importance to the whole field (see caption of Fig. 5 for more details).

### Data acquisition for the overview of current research projects

To obtain an overview on the research landscape and third-party funding, we pursued two complementary strategies. First, we sent an email (along with two reminders) to the Directors of all German Departments of Psychiatry and Psychotherapy at Universities or University Hospitals (i.e., the members of LIPPs e.V.; www.uni-lipps.de). We asked for information about current research projects on bipolar disorder (title, PI, content, funding agency, international collaboration) with emphasis on projects funded by German agencies (i.e., German Research Foundation, DFG; Federal Ministry of Education and Research, BMBF) as well as the European Union (EU). Thereby, 43 Departments from 39 Universities could be reached, of which 20 Universities (51.3%) responded. Only currently running projects were considered. Also, we screened publicly available databases of German and European funding measures: *gepris* for DFG funding (https://gepris.dfg.de/), the internal BMBF search engine for the topic “health” (https://www.bmbf.de/bmbf/de/forschung/gesundheit/gesundheit_node.html) and *cordis* for EU projects (https://cordis.europa.eu/projects); for the latter, only projects with a German partner were considered further. As a search string, only the word “bipolar” was set to retrieve as many hits as possible; all descriptions were then manually screened to restrict the findings to projects that were on “bipolar disorder” in a narrower sense. Thereby, projects on completely different topics (e.g., physics, histology or the retina) were excluded, as well as a few projects that had only very loose ties to bipolar disorder (e.g., electrophysiological examinations on a channel protein that has, *inter alia*, been nominated as being encoded by a gene associated with bipolar disorder). The latter category however comprised less than 10 projects. Projects from two further Universities were identified (corresponding to one EU project) using this strategy. Only currently ongoing projects were considered.

## Results

### Descriptive information

**Fig. 1:**
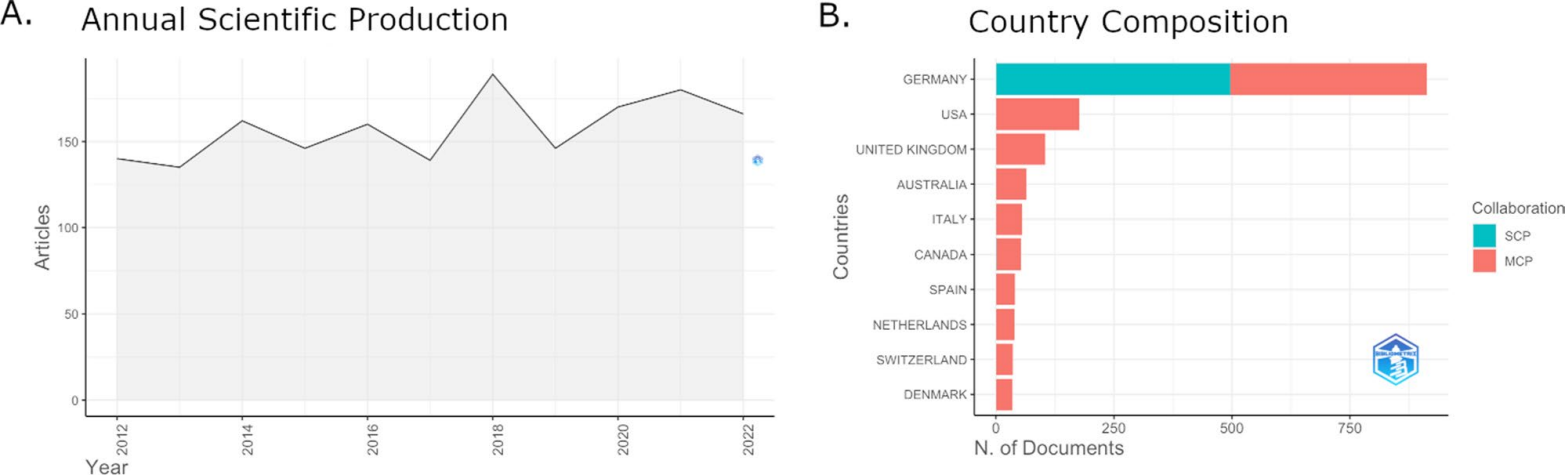
Scientific production. Annual scientific production under German collaboration remained relatively stable during the last decade (**A**). More than 50% of articles were published with a corresponding author seated in Germany. The remaining articles originated in international collaboration projects with the corresponding author most frequently being seated in the USA, UK, Australia, Italy, and Canada (**B**). *Note*: SCP = Single Country Publications, MCP = Multiple Country Publications, N.= Number

Of the 1,763 publications on bipolar disorder, which were included in the analysis, 1,415 were regular (21 early access) and 348 were review (9 early access) articles. Figure 1A illustrates the number of annual publications during the last decade of bipolar disorder research in Germany. The annual growth rate was 1.7%. Accordingly, German productivity in the field remained relatively constant for the whole decade, with no substantial increase or decrease concerning productivity. Figure 1B shows the number of articles partitioned according to the country of its corresponding author at the time of publication. 913 (51.8%) of the articles under German collaboration were indeed published by a corresponding author seated in Germany, of which 497 (54.4%) were single country publications (i.e., **only** German authors were involved in its production). Accordingly, a substantial part of publications with a German corresponding author had an international orientation (*N* = 416, 45.6%). The remaining 850 articles were collaboration projects with German authors headed by corresponding authors with affiliations in the USA (*N* = 177), United Kingdom (*N* = 177), Australia (*N* = 65), Italy (*N* = 55), Canada (*N* = 53), Spain (*N* = 40), Netherlands (*N* = 39), Switzerland (*N* = 36), and Denmark (*N* = 35).

### Analysis of the scientific network

#### Analysis of the most productive institutions

**Fig. 2:**
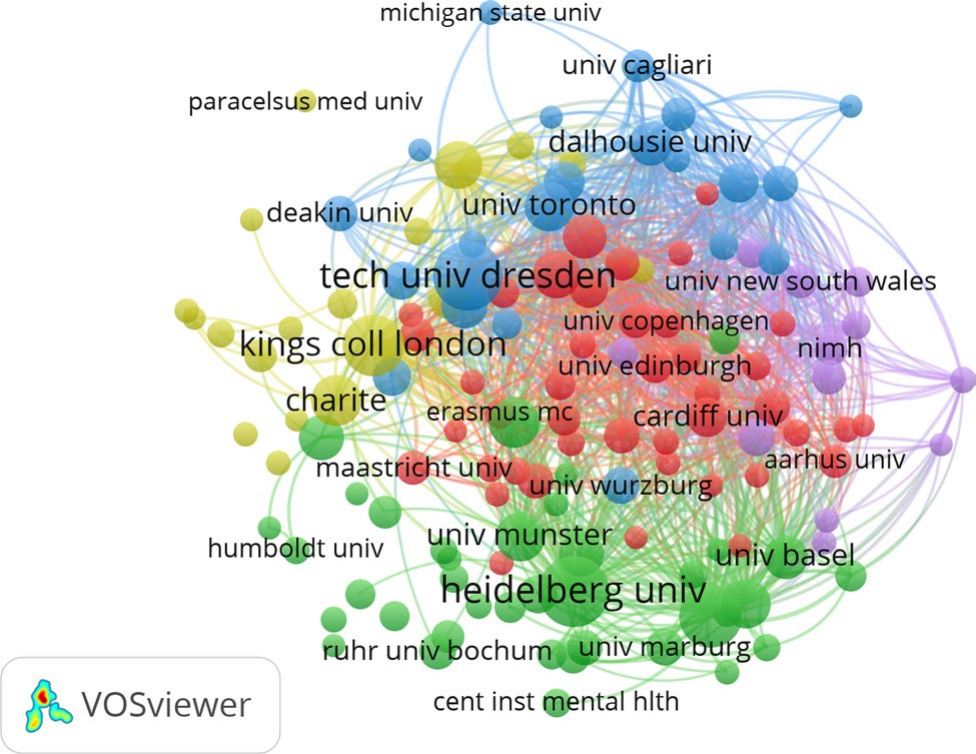
Collaboration network of most productive institutions. While two of the clusters – cluster 1 (depicted in red) and cluster 3 (depicted in blue) – were dominated by institutions seated in foreign countries, cluster 2 (depicted in green) connects a large number of German institutions. *Note*: Institutions that have published more than 25 publications in the database were included in this visualization

In total, 3,355 different institutions were involved in production of the present publications. Five of the ten most frequently indicated (i.e., most productive) institutions were seated in Germany (N refers to their total number of mentions): University of Bonn (*N* = 355), Heidelberg University (including the Central Institute for Mental Health, ZI Mannheim; *N* = 321), Technical University Dresden (*N* = 288), University of Münster (*N* = 164) and Ludwig Maximilian University of Munich (*N* = 154). The other five were seated abroad, i.e., Kings College London (*N* = 315), University of Melbourne (*N* = 201), University of Toronto (*N* = 192), Karolinska Institute (*N* = 181), and University of Barcelona (*N* = 146). When analyzing the network structure of institutions that produced more than 25 publications within the last decade, five major clusters emerged (see Fig. 2; Table 1 for a full description of Cluster members).

**Table 1:** Institutional collaboration network

**Cluster 1 (42 items)**		**Depicted in red**	
Aarhus University	DEN	Broad institute of MIT & Harvard	USA
Cardiff University	UK	Columbia University	USA
Copenhagen University Hospital	DEN	Erasmus University Medical Center	NL
Erasmus University	NL	Harvard Medical School	USA
Harvard University	USA	Icahn School of Medicine at Mount Sinai	USA
Karolinska Institute	SWE	Maastricht University	NL
Massachusetts General Hospital	USA	University of Galway	IRE
Oslo University Hospital	NOR	Radboud University Nijmegen	NL
Stanford University	USA	SUNY Upstate Medical University	USA
Trinity College Dublin	IRL	University College London	UK
University of Bergen	NOR	University of California Irvine	USA
University of Chicago	USA	University of Copenhagen	DEN
University of Edinburgh	UK	University of Gothenburg	SWE
University of Groningen	NL	University of Iowa	USA
University Medical Center Utrecht	NL	**Greifswald University Hospital**	**DEU**
University of Michigan	USA	University of North Carolina	USA
University of New Mexico	USA	University of Oslo	NOR
University of Pennsylvania	USA	University of Pittsburgh	USA
University of Queensland	AUS	University of Southern California	USA
University of Sydney	AUS	Vrije Universiteit Amsterdam	NL
Washington University in St. Louis	USA	Yale University	USA
**Cluster 2 (37 items)**		**Depicted in green**	
**Central Institute of Mental Health Mannheim**	**DEU**	**Charité University Hospital Berlin**	**DEU**
FondaMental foundation	FRA	**Free University of Berlin**	**DEU**
**German Center for Neurodegenerative Diseases**	**DEU**	**Goethe University Frankfurt**	**DEU**
**Heidelberg University**	**DEU**	**Humboldt University of Berlin**	**DEU**
**Jena University hospital**	**DEU**	**Johannes Gutenberg University Mainz**	**DEU**
**Ludwig Maximilian University of Munich**	**DEU**	**Max Planck Institute for Human Cognitive & Brain Sciences**	**DEU**
**Max Planck Institute of Psychiatry**	**DEU**	Medical University of Graz	AUT
Otto von Guericke University Magdeburg*	**DEU**	Philipps University Marburg*	**DEU**
Juelich Research Center*	**DEU**	**Ruhr University Bochum**	**DEU**
**Technical University Munich**	**DEU**	University of Basel	CH
University of Bern	CH	**University of Bonn**	**DEU**
University of Cambridge	UK	**University of Duisburg-Essen**	**DEU**
**University of Göttingen**	**DEU**	**University Hospital of Bonn**	**DEU**
**University of Leipzig**	**DEU**	**University Medical Center Göttingen**	**DEU**
**University Medical Center Hamburg**	**DEU**	**University of Munich**	**DEU**
**University of Münster**	**DEU**	**University of Tübingen**	**DEU**
**University of Ulm**	**DEU**	University of Zürich	CH
**Cluster 3 (22 items)**		**Depicted in blue**	
Aarhus University Hospital	DEN	Aristotle University of Thessaloniki	GRC
Dalhousie University	CAN	Deakin University	AUS
**Goethe University of Frankfurt**	**DEU**	Mayo Clinic	USA
Michigan State University	USA	Mood disorders Centre of Ottawa	CAN
Poznan University of Medical Sciences	POL	**Dresden University of Technology**	**DEU**
University of Adelaide	AUS	University in Cagliari	ITA
University of Calgary	CAN	University of California, Los Angeles	USA
University of California, San Diego	USA	**University of Cologne**	**DEU**
University of Helsinki	FIN	University of Melbourne	AUS
University of Paris-Est Sup	FRA	University of Sao Paulo	BRA
University of Toronto	CAN	**University of Würzburg**	**DEU**
**Cluster 4 (19 items)**		**Depicted in yellow**	
**Charité University Hospital Berlin**	**DEU**	Centre for Addiction and Mental Health	CAN
Zucker School of Medicine	USA	Feinstein Institutes for Medical Research	USA
**Hannover Medical School**	**DEU**	Katholieke Universiteit Leuven	NL
Kings’s College London	UK	Newcastle University	UK
Northwell Health	USA	**Paracelsus Medical Private University**	**DEU**
South London & Maudsley NHS Foundation Trust	UK	University of Barcelona	ESP
The University of British Columbia	CAN	University of Cincinatti	USA
**University of Freiburg**	**DEU**	**University of Lübeck**	**DEU**
University of Ottawa	CAN	University of Oxford	UK
Zucker Hillside Hospital	USA		
**Cluster 5 (12 items)**		**Depicted in purple**	
Alexandru Obregia Clinical Psychiatric Hospital	ROU	National Institute of Health and Medical Research	FRA
Johns Hopkins University	USA	McGill University	CAN
**Munich Cluster for Systems Neurology**	**DEU**	National Institute of Mental Health	USA
Neuroscience Research Australia	AUS	**Frankfurt University Hospital**	GER
University of Liverpool	UK	University of New South Wales	AUS

Note: Institutions marked with an asterisk have been mentioned twice (within the same cluster) due to orthographic differences in data base records. Country codes were specified in ISO-3166-1 convention. German institutions are indicated in bold

#### Analysis of the most productive authors and sources

**Fig. 3:**
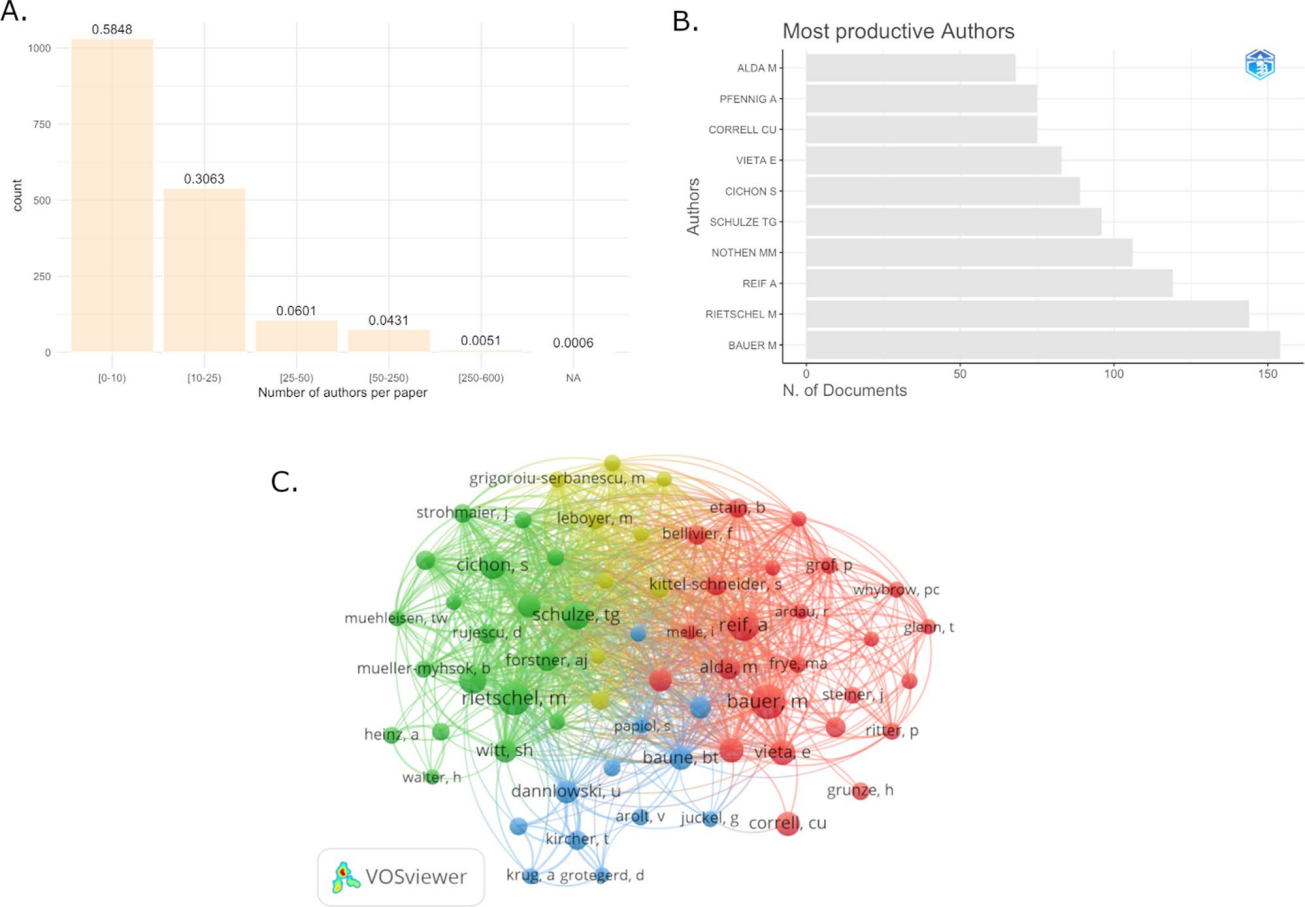
Author contributions. Most of the published articles were original scientific papers (**A**). Eight of the most productive corresponding authors were affiliated in Germany at least for a substantial part of the time accounted for in this analysis (**B**). The collaboration network of authors that published more than 25 publications in the last decade contained four major clusters, which were all highly interconnected (**C**)

In total, 11,253 authors contributed to the included articles. While only 25 articles were written by a single author, the remaining 1,738 articles were written under collaboration of several authors (mean = 15.5, SD = 32.6, max = 571). Of these, 58.5% (*N* = 1,031) were original scientific papers authored by 2–9 authors, 36.6% (*N* = 646) were team works authored by 10–49 authors and 4.9% (*N* = 86) were multi-center studies with more than 50 authors (Fig. 3A). The 10 most productive authors, or research teams led by the respective author, were Michael Bauer (TU Dresden, *N* = 154, Total number of citations (TC) = 7,981, h-index = 38), Marcella Rietschel (UMM Mannheim, *N* = 144, TC = 8,238, h-index = 41), Andreas Reif (University Hospital Frankfurt, *N* = 119, TC = 5,910, h-index = 29), Markus M. Nöthen (University of Bonn, *N* = 106, TC = 6,040, h-index = 34), Thomas G. Schulze (LMU Munich, *N* = 96, TC = 6,989, h-index = 33), Sven Cichon (Research Center Jülich, *N* = 89, TC = 6,963, h-index = 38), Eduard Vieta (University of Barcelona, *N* = 83, TC = 4,491, h-index = 31), Christoph U. Correll (Charité Berlin, *N* = 75, TC = 1,573, h-index = 22), Andrea Pfennig (TU Dresden, *N* = 75, TC = 2,909, h-index = 27), and Martin Alda (Dalhousie University, *N* = 68, TC = 4028, h-index = 29).^1^ See Fig. 3B. The collaboration network of authors that published more than 25 publications in the last decade (Fig. 3C) consists of four clusters, which are all highly interconnected. See Table 2 for a full description of cluster members in the collaboration network.

**Table 2:** Author collaboration network

Author	*N*	Affiliation	Country
**Cluster 1 (24 items)**		**Depicted in red**	
Alda, Martin	68	Dalhousie University	CAN
Andreassen, Ole	65	University of Oslo	NOR
Ardau, Raffaella	30	University of Cagliari	ITA
Bauer, Michael	154	University Hospital Leipzig	DEU
Bellivier, Frank	47	University Hospital Paris	FRA
Berk, Michael	52	Deakin University	AUS
Correll, Christoph U.	74	Charité Berlin	DEU
Del Zompo, Maria	31	University of Cagliari	ITA
Etain, Bruno	49	University Paris Cité	FRA
Frye, Mark A.	36	Mayo Clinic	USA
Glenn, Tasha	30	University of Technology Dresden	DEU
Grof, Paul	36	University of Ottawa	CAN
Grunze, Heinz	41	Paracelsus Medical Private University	DEU
Kittel-Schneider, Sarah	58	University of Würzburg	DEU
Lewitzka, Ute	30	University of Technology Dresden	DEU
Melle, Ingrid	31	University of Oslo	NOR
O’Donovan, Claire	30	Dalhousie University	CAN
Pfennig, Andrea	75	University of Technology Dresden	DEU
Reif, Andreas	119	University Hospital Frankfurt	DEU
Ritter, Philipp	37	University of Technology Dresden	DEU
Severus, Emanuel	35	University of Technology Dresden	DEU
Steiner, Johann	38	Otto von Guericke University Magdeburg	DEU
Vieta, Eduard	83	University of Barcelona	ESP
Whybrow, Peter C.	33	University of California, Los Angeles	USA
**Cluster 2 (19 items)**		**Depicted in green**	
Cichon, Sven	89	University of Basel	CHE
Degenhardt, Franziska	67	University of Bonn	DEU
Forstner, Andreas J.	58	University of Bonn	DEU
Heinz, Andreas	37	Charité Berlin	DEU
Herms, Stefan	36	University of Bonn	DEU
Hoffmann, Per	39	University of Bonn	DEU
Maier, Wolfgang	30	University of Bonn	DEU
Mattheisen, Manuel	47	Ludwig Maximilian University of Munich	DEU
Meyer-Lindenberg, Andreas	40	Central Institute of Mental Health Mannheim	DEU
Mühleisen, Thomas W.	33	University of Basel	CHE
Müller-Mysok, Bertram	37	Max Planck Institute of Psychiatry	DEU
Nöthen, Markus M.	95	University of Bonn	DEU
Rietschel, Marcella	143	University Medical Centre Mannheim	DEU
Rujescu, Dan	47	Medical University of Vienna	AUS
Schulze, Thomas G.	96	Ludwig Maximilian University of Munich	DEU
Streit, Fabian	33	Central Institute of Mental Health Mannheim	DEU
Strohmaier, Jana	43	University Medical Centre Mannheim	DEU
Walter, Henrik	30	Charité Berlin	DEU
Witt, Stephanie		University Medical Centre Mannheim	DEU
**Cluster 3 (12 items)**		**Depicted in blue**	
Arolt, Volker	36	University of Münster	DEU
Baune, Bernhard T.	65	University of Münster	DEU
Dannlowski, Udo	66	University Hospital Münster	DEU
Falkai, Peter	59	Ludwig Maximilian University of Munich	DEU
Grotegerd, Dominik	32	University of Münster	DEU
Gruber, Oliver	37	University of Heidelberg	DEU
Heilbronner, Urs	32	Ludwig Maximilian University of Munich	DEU
Juckel, Georg	35	Ruhr University Bochum	DEU
Kircher, Thilo	47	Philipps University Marburg	DEU
Krug, Axel	34	Philipps University Marburg	DEU
Nenadic, Igor	47	Philipps University Marburg	DEU
Papiol, Sergi	47	Ludwig Maximilian University of Munich	DEU
**Cluster 4 (9 items)**		**Depicted in yellow**	
Fullerton, Jance M.	32	University of New South Wales	AUS
Grigoroiu-Serbanescu, Maria	53	Obregia Psychiatry Hospital Bucharest	ROU
Jamain, Stéphane	35	University Paris-Est: Créteil	FRA
Landén, Mikael	43	University of Gothenburg	SWE
Leboyer, Marion	47	University Paris-Est: Créteil	FRA
McMahon, Francis	32	National Institute of Mental Health	USA
Mitchell, Philip B.	44	University of New South Wales	AUS
Potash, James B.	31	Johns Hopkins University School of Medicine	USA
Schofield, Peter R.	34	University of New South Wales	AUS

Note: N refers to the number of documents in the data base. Country codes were specified in ISO-3166-1 convention

Articles were published in 420 journals with the ten most important sources being highly regarded (IF = WOS impact factor 2022 as retrieved from https://www.scijournal.org): Journal of Affective Disorders (*N* = 83, IF = 4.8), Translational Psychiatry (*N* = 63, IF = 6.2), European Archives of Psychiatry and Clinical Neuroscience (*N* = 58, IF = 5.3), International Journal of Bipolar Disorders (*N* = 58, IF = 4.3), Bipolar Disorders (*N* = 57, IF = 6.7), Frontiers in Psychiatry (*N* = 53, IF = 4.1), Molecular Psychiatry (*N* = 53, IF = 16.0), Psychiatry Research (*N* = 44, IF = 3.2), Schizophrenia Research (*N* = 40, IF = 4.9), and Journal of Psychiatric Research (*N* = 37, IF = 4.8). (*Note*: An additional short description of the twenty most cited articles which were referred to in the publications can be found in the supplementary material).

### Conceptual structure – analysis of research topics

#### Co-word analysis

**Fig. 4:**
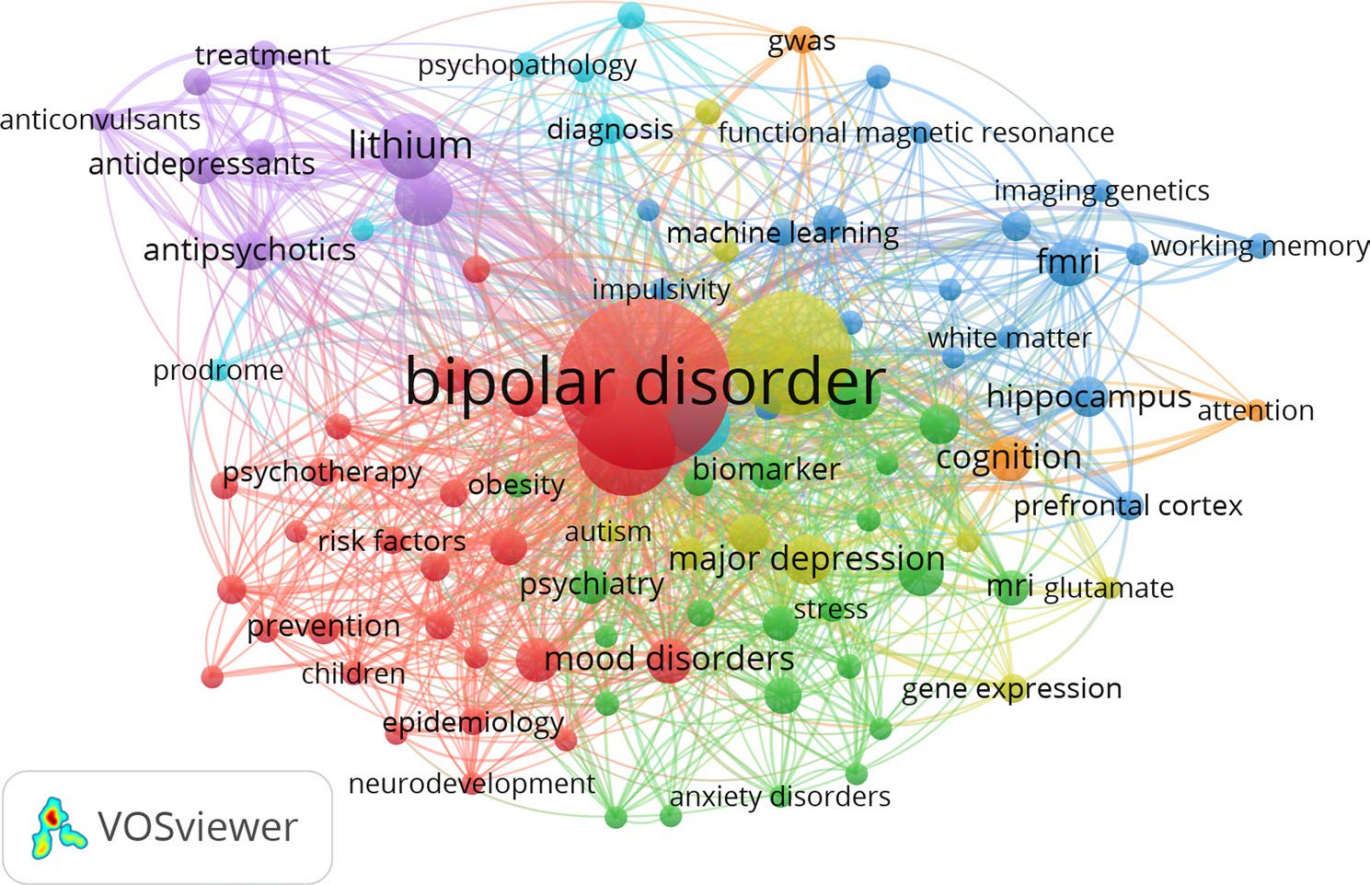
Co-word analysis network. While the Cluster 1 (depicted in red) is concerned with early recognition and therapeutical interventions, Clusters 2 (depicted in green) and 3 (depicted in green) are rather concerned with neuroimaging methods, Clusters 4 (depicted in yellow) and 7 (depicted in orange) are dominated dominated by genetic studies, Cluster 5 (depicted in purple) with pharmacological interventions and Cluster 6 (depicted in turqouise) with diagnosis and differentiation from psychosis. *Note*: The size of the nodes reflect the occurrence frequency of the respective keyword, thickness of the connecting line represents the strength of association

As has to be expected by the method of the literature research, the most frequently indicated keyword in the data base was bipolar disorder OR bipolar disorders (*N* = 605). This was followed by schizophrenia (*N* = 303), depression OR major depressive disorder OR major depression (*N* = 297), lithium (*N* = 86), psychosis (*N* = 79), mania (*N* = 66), fMRI OR functional magnetic resonance imaging (*N* = 53), cognition (*N* = 42), genetics (*N* = 42), and meta-analysis (*N* = 42). Clustering analysis of the co-occurence network of keywords that were mentioned at least ten times in the data base revealed seven major clusters (Fig. 4). An interpretation of the co-occurrence network suggests, that one large cluster (cluster 1, depicted in red) was concerned with early recognition and therapy of bipolar disorders, two of the clusters (cluster 2 and 3, depicted in blue and green) with neuroimaging, two of the clusters (cluster 4 and 7 depicted in yellow and orange) with (functional) genetics/genomics, one cluster (cluster 5 depicted in purple) with pharmacological interventions, and one cluster with (early) diagnosis (cluster 6 depicted in turqouise). The multitude of connections between the three clusters indicates, however, that a strict thematic separation of topics would be rather artificial. See Table 3 for a full list of keywords within each cluster.

**Table 3:** Cluster members of the co-word analysis

Keyword	*N*		*N*
**Cluster 1**		**Depicted in red**	
adhd	25	adolescents	17
anxiety	37	bipolar disorder*	605
children	12	comorbidity	27
depression	189	early intervention	12
early recognition	18	epidemiology	16
insomnia	11	mental disorders	18
mental health	16	meta-analysis	42
mood disorders	41	neurodevelopment	11
pharmacotherapy	13	prevalence	12
prevention	21	psychotherapy	18
psychotic disorders	11	quality of life	16
risk factors	15	severe mental illness	10
sleep	11	systematic review	15
**Cluster 2 (22 items)**		**Depicted in green**	
anxiety disorders	11	BDNF	11
biomarkers*	38	bipolar	25
cortical thickness	11	epigenetics	16
genetics	42	inflammation	29
major depressive disorder	58	mental illness	11
metabolic syndrom	12	mood disorder	10
MRI	25	neuroimaging	32
neuroinflammation	11	obesity	14
oxidative stress	12	psychiatric disorders	18
psychiatry	29	review	11
stress	13		
**Cluster 3 (18 items)**		**Depicted in blue**	
amygdala	18	CACNA1C	11
diffusion tensor imaging	10	emotion regulation	14
fMRI*	53	functional connectivity	12
hippocampus	32	imaging genetics	10
impulsivity	10	machine learning	16
magnetic resonance imaging	23	prefrontal cortex	18
social cognition	11	transdiagnostic	12
voxel-based morphometry	10	white matter	12
working memory	13		
**Cluster 4 (10 items)**		**Depicted in yellow**	
affective disorders	34	autism	11
dopamine	14	gene expression	17
glutamate	11	major depression	50
microglia	11	schizophrenia	303
serotonin	12	suicide	21
**Cluster 5 (8 items)**		**Depicted in purple**	
anticonvulsants	10	antidepressants	26
antipsychotics	31	bipolar depression	18
lithium	86	mania	66
mood stabilizers	15	treatment	17
**Cluster 6 (7 items)**		**Depicted in turquoise**	
affective disorder	11	diagnosis	20
prediction	11	prodrome	10
psychopathology	13	psychosis	79
schizoaffective disorder	15		
**Cluster 7 (3 items)**		**Depicted in orange**	
attention	11	cognition	42
gwas	16		

Note: Keywords marked with an asterisk have been mentioned twice (within the same cluster), due to orthographic differences in data base records; N refers to number of occurrences

#### Thematic map of author keywords

**Fig. 5:**
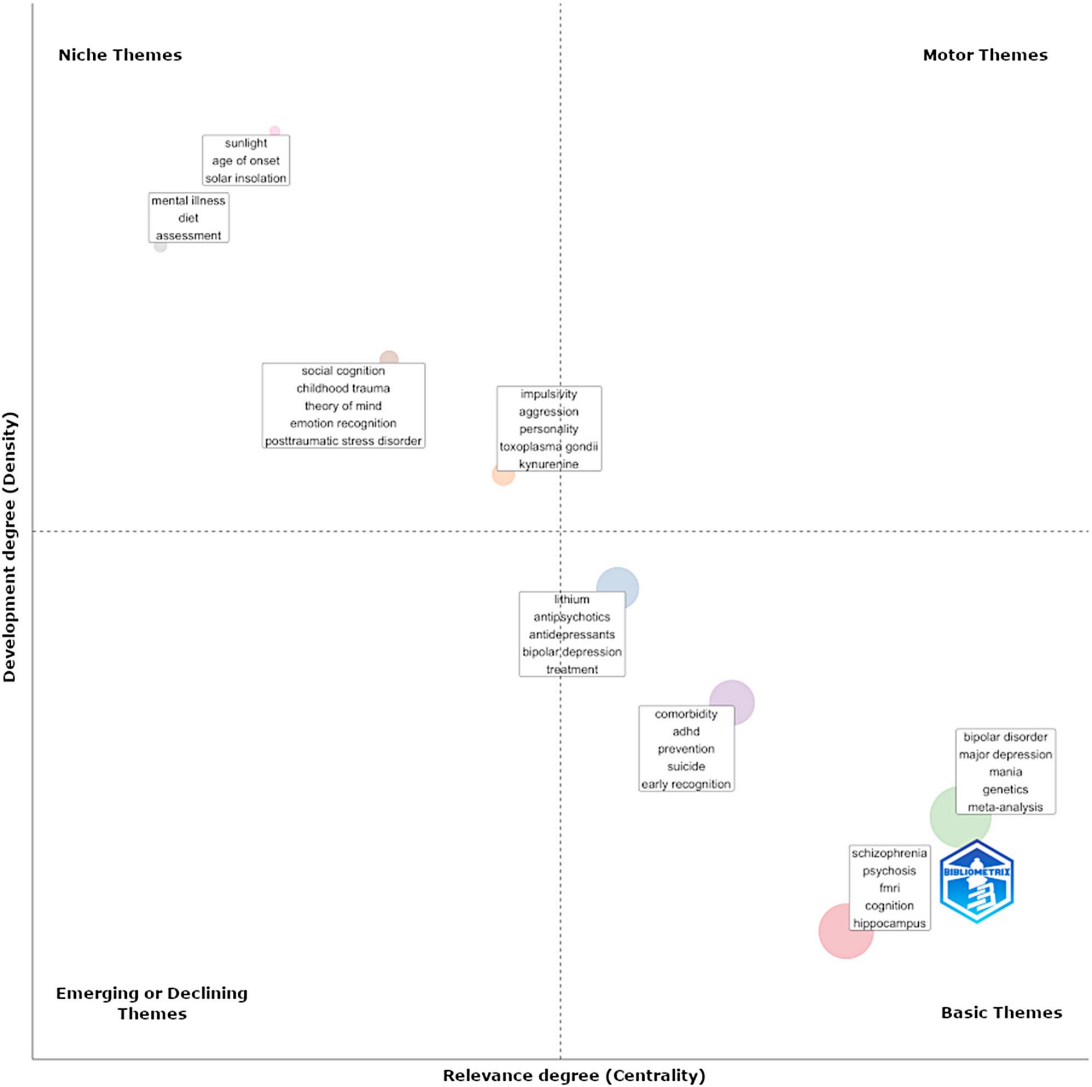
Thematic map of author keywords. The thematic map of the field was characterized by *basic themes*, that are important for the whole community without showing a high cohesiveness (lower-right quadrant) and *niche themes*, that are highly developed but isolated to specific research sub-communities, i.e. without a high number of relations with other themes. *Note*: The thematic map uses sub-group analysis to identify specific themes that are prominent in the field and plots them in a thematic diagram based on Callon density (as a measure of the topic’s cohesiveness and thus development) and Callon centrality (as a measure of the degree of correlation with other topic’s and thus importance for the whole field). Four topologies of themes are plotted in four different quadrants: *Motor themes* (upper-right quadrant) are characterized by high centrality and high density. These themes are highly developed and important for the whole research field. *Basic themes* (lower-right quadrant) are high in centrality and low in density. These themes are thus important for the whole research field, even though the topics themselves are not highly developed (yet). *Emerging / declining themes* (lower-left quadrant) are low in centrality and low in density. Accordingly, these themes are both, weakly developed and marginal. Lastly, *niche themes* (upper-left quadrant) are highly developed, but isolated, i.e. they are characterized by well developed internal links (high density) but unimportant external links (low centrality)

Several themes were identified by analyzing the provided author keywords. No clear motor theme could be identified, i.e., a theme that is important to and well established within the research field. However, some basic themes emerged, that were important for the research field, even though they were not highly developed, yet. Four clusters of basic themes could be identified, of which ‘schizophrenia, psychosis, fMRI, cognition, and hippocampus’ was of lowest density and thus a rather developing theme. The other three clusters were in the ‘middle field’ of thematic development – ‘bipolar disorder, major depression, mania, genetics, meta-analysis’, ‘comorbidity, ADHD, prevention, suicide, early recognition’, and ‘lithium, antipsychotics, antidepressants, bipolar depression, treatment’. Besides these basic themes, several niche themes were identified: ‘impulsivity, aggression, personality, toxoplasma gondii, kynurenine’, ‘social cognition, theory of mind, childhood trauma, social cognition’, ‘mental illness, diet, assessment’, and ‘sunlight, age of onset, solar insolation’. Those themes are highly developed but rather isolated within the research field (Fig. 5).

### Grant support of projects on bipolar disorder

Seven out of the 20 institutions responding to our survey explicitly stated that none of their research projects focused on bipolar disorders. There were a few projects that accounted for the majority of research activities: the BMBF-funded BipoLife consortium (bipolife.org), which however ran out in 2022 and thus cannot be counted in the present analysis; the same holds true for the EU-funded project Moodinflame (moodinflame.eu; Universities of Münster and Ulm).

A major current research structure on mood disorders is the Research Group FOR 2107, funded by the DFG (for2107.de), which revolves around the long-term course of affective disorders, not specific to but including bipolar disorder. The FOR2107 (ending in 2023) connects the Universities of Marburg (lead), Münster, Kiel, Bonn and the Central Institute for Mental Health Mannheim. It gave rise to three following individual grants by the DFG, all to PIs at the University of Münster. Four other collaborative research efforts by the DFG, RTG2862, iRTG2150, FOR2858 and iRTG2773, which had a transdiagnostic approach, had one subproject each that also included – amongst others – research questions on bipolar disorder. The Heisenberg-professorship, awarded to PI Hahn at the University of Münster, also supports research on bipolar disorder. Finally, there is one individual grant to PI Noethen at University of Bonn that includes bipolar disorder research. To conclude, apart from FOR 2107 (and its follow-ups), which has one research focus on bipolar disorder, there are only very few (six) research projects on bipolar disorder funded by the DFG.

Since the collaborative project “Bipo-Life” ran out, there is no national funding on bipolar disorder from the BMBF. In 2023, the German Center for Mental Disorders (DZPG) was founded, which is co-financed by the BMBF and which also includes research on bipolar disorders. However, the extent to which bipolar disorder is considered in the DZPG is as yet unclear. Only three of the centers that formed BipoLife are also members of the DZPG (Berlin, Munich, Tübingen). The collaborative BMBF project COMMITMENT includes, amongst others, research questions on bipolar disorder in one bioinformatic subproject. There are four collaborative projects on the European level, which are administered by the BMBF and focus on bipolar disorder. These are Plot-BD (PerMed framework), DynAMoND (EraNet Neuron framework), BIPCOM (PerMed framework), and UNMET (EraNet Neuron framework).

Two large-scale European Horizon 2020 projects on bipolar disorder have contributions from Germany, R-LiNK (ending in 2023) and PsychSTRATA. Intramural research projects were reported by six institutions. All members but one of the National Center of Affective Disorders (NCAD; www.ncad.health) reported research on bipolar disorders funded by either DFG and/or BMBF.

## Discussion

Bibliometric analyses permit an overview over the intellectual and conceptual structure of the scientific literature of a specific time period and / or a specific country. Our bibliometric analysis of the last decade revealed a vivid landscape of German research on bipolar disorder that was characterized by well-networked, international projects mainly dealing with psychiatric genetics, neuroimaging and early prevention studies. However, in striking contrast to the high burden of disease and its enormous socioeconomic consequences, current grant support for research on bipolar disorders is surprisingly low.

### Intellectual structure of German research on bipolar disorder

Analysis of the intellectual structure of German research on bipolar disorders revealed large research networks with a highly international orientation: German scientists were involved in a large number of publications written by corresponding authors from abroad. Furthermore, about 50% of the articles that were written under German leadership (with a German corresponding author) had an international orientation as well, i.e. were written in cooperation with foreign institutions. In line with earlier bibliometric analyses (López-Muñoz et al. 2006; Vogelzang et al. 2012; Zakaria et al. 2023), country as well as institutional collaboration analysis revealed that associations were mainly built with highly developed countries of the global North as USA, UK and Australia. In contrast, no Chinese or Indian institution was mentioned among the most important institutions even though China is one of the most productive scientific nations and India fosters a vivid culture of research on bipolar disorder (Grover et al. 2021).

Further network analyses specified collaborations between institutions with a five-cluster solution (compare Fig. 2; Table 1): While cluster 1 (depicted in red) was dominated by international institutions with the University of Greifswald as the only German member of the cluster, cluster 2 (depicted in green) reflected tight cooperation between several German institutions^2^ with only some connections to France, Austria, Switzerland and the United Kingdom. The remaining three clusters were characterized by sub-networks that probably follow specific thematic orientations, i.e., the University Hospital Frankfurt, Technical University Dresden, University of Cologne and University of Würzburg orchestrating international cooperation in Cluster 3 (depicted in blue), Charité University Hospital Berlin, Hannover Medical School, Paracelsus Medical Private University, University of Freiburg, and University of Lübeck orchestrating international cooperation in Cluster 4 (depicted in yellow) and again University Hospital Frankfurt together with the Munich Cluster for Systems Neurology being central to Cluster 5 (depicted in purple). Part of this vibrant network landscape is also covered by the ten most productive authors, as those were amongst others also seated in Dresden, Frankfurt, Munich, and Berlin. However, it should be noted for both techniques (i.e., most productive institutions and authors), that these methods are purely quantitative and may therefore lead to a bias in favor of large, financially strong institutes with a particular interest in bipolar disorder. Although this provides important information on the intellectual structure of the German research landscape, it does not consider the many smaller research groups that also make an important contribution to bipolar disorder research.

A ‘working style’ of large, internationally oriented networks was also reflected in the authorship of publications – more than 30% of publications were team works by 10 to 50 authors and approximately 5% of the publications were large multi-center studies with more than 50 co-authors – probably due to the high occurrence of genetic and neuroscientific ‘big data’ studies. This impression is in line with the ten most important sources in which 546 of the articles were published. While some of these sources (as e.g. *International Journal of Bipolar Disorders*) are highly regarded scientific journals dealing with the topic for affective disorders, some others were methodically specific journals with an orientation towards neurobiology (as e.g. *Molecular Psychiatry*) in which such multi-center work might have been published.

### Conceptual structure of German research on bipolar disorders

In a former bibliometric analysis covering research that has been published at least two decades ago, around 40% of publications focused on pharmacotherapy of bipolar disorder (López-Muñoz et al. 2006). A couple of years later the additional topic of psychiatric genetics was mentioned as emerging theme in the work of the most prolific authors engaged in research of bipolar disorder (Vogelzang et al. 2012). Our current data further supports a change in thematic trends in favor of psychiatric genetics, as several of our analysis strains indicated a thematic dominance of this topic in German research activity of the last decade: journals with a specific orientation towards molecular studies (as e.g. *Molecular Psychiatry* and *Translational Psychiatry*) were among the ten most important sources. Furthermore, genetic topics were represented in four out of seven keyword clusters of the co-word analysis (compare Fig. 4), i.e., ‘epigenetics’ and ‘genetics’ in cluster 2 (depicted in green), ‘imaging genetics’ in cluster 3 (depicted in blue), ‘gene expression’ in cluster 4 (depicted in yellow) and ‘GWAS’ in cluster 7 (depicted in orange). Neuroimaging studies as well as research on risk factors and early recognition of the disease were further central themes in research on bipolar disorder. Studies on lithium dominated the field on pharmacotherapy, however, comparably little studies were on other medications, psychotherapy, neuromodulation or other modalities. This is backed up by data on publicly funded clinical studies in Germany: not a single clinical study on bipolar disorder could be identified in the Clinical Study program of the DFG/BMBF. These findings are worrisome, given that bipolar disorder affects 1–2% of the adult population. In conjunction with the fact that the pharmaceutical industry only runs very few trials on bipolar disorder, this leaves little hope for the many people suffering this disabling condition. This finding prompts more efforts to develop new treatments for bipolar disorder and to provide according funding for corresponding research projects.

### Future directions in German research activity on bipolar disorders

Personal communications with leading German researchers in the field of bipolar disorder revealed that on-going and future projects will continue some of the thematic clusters that have been identified in our conceptual analysis. Two large research networks dominate current research on the topic: The **BMBF research association * BipoLife*** (under collaboration of the universities of Frankfurt, Tübingen, Heidelberg and Marburg, TU Dresden and Charité Berlin) focusses on early recognition and therapeutical intervention in high-risk populations and first-episode patients (compare cluster 1 in the co-word analysis). This collaborative research project however ran out in 2022 and, despite being highly successful, was not continued. The **DFG project FOR 2107** (under collaboration of the universities of Marburg, Münster, Giessen, and Bonn and the Central Institute of Mental Health Mannheim) investigates the influence of environmental, neurobiological and genetic factors in the development of affective disorders (as major depression and bipolar disorder) and their long-term trajectories (compare cluster 2 in the co-word analysis). FOR 2107 ran out last year (2023) and gave rise to three further, smaller follow-up projects awarded to individual PIs at the contributing centers. Also, a transregio CRC focusing on mood disorders including bipolar disorder has been proposed, which however has not yet been decided upon. Beyond that, R-LiNK (on the prediction of lithium response; the follow-up project SALTED is currently under review), BIPCOM (on the prevalence of metabolic syndrome in bipolar disorder) and PsychSTRATA (on escalating treatment of severe mental illness) are European projects where more than one institution participates. While they revolve around treatment of the disorder in one or the other way, comparable to current German research funding (see above), none of them is on novel treatments.

### Research funding in Germany

While the bibliometric analysis shows very productive German research on bipolar disorder in the recent past, which was excellently connected especially to the global North, current funding by public agencies falls short. Landmark funding was only provided once, by the BMBF funded collaborative project “BipoLife” which focused on early recognition, psychotherapy and the use of digital biomarkers. The other large network, FOR2107 (see above) did not specifically focus on bipolar disorder while summarizing it under “affective disorder” (most of the research in FOR2107 was on unipolar depression). Apart from these collaborative efforts, there is little to none public funding for research on bipolar disorder in Germany. If all DFG and BMBF projects given in the results section are summed up, the sum of annual public funding in Germany is maximally around one million Euro. This number would increase to around 1.4 Mio € if the co-funded Era PerMed and EraNet Neuron projects are taken into account. Still, this number is stunningly low given the prevalence of bipolar disorder and the annual cost of the disorder, estimated at direct mental healthcare cost of around 5,000€ per capita per year (Gustavsson et al. 2011; Kleine-Budde et al. 2014) (i.e., around 2,500 Mio per year in Germany, conservatively assuming a prevalence of 1% in the adult population). One might argue that we missed funded projects due to our search strategy; however, as there was a high overlap between the institutions and authors providing most of the research output and the institutions listed in the *gepris*, BMBF, and *cordis* databases, it is unlikely that many projects were missed.

## Conclusions

The German research landscape on bipolar disorder is quite productive and centered around few research hubs; psychiatric genetics is one of the core fields, as is neuroimaging, risk factors and early recognition. Around half of the research output is done within the context of international collaborations, especially with the global North. However, there are comparably few studies on novel treatments. This is reflected by the amount of public funding: currently, not a single clinical trial on bipolar disorder is funded by national research agencies. Overall, the amount of funding which is directed at bipolar disorders is very low, which is worrisome given the prevalence and burden of bipolar disorder. We therefore urgently call for an increase in funding allocated to bipolar disorder, to improve the lives of many patients as well as their relatives.

### Electronic supplementary material

Below is the link to the electronic supplementary material.

Supplementary Material 1

Supplementary Material 2

## Data Availability

Data underlying the bibliometric analysis has been uploaded as supplementary material.

## Footnotes

1 *Note*: Sven Cichon and Christoph U. Correll spent part of the analyzed period abroad (Switzerland, USA); Eduard Vieta and Martin Alda were seated in Spain and Canada, respectively, for the whole period.

2 *Note*: Some of the involved institutions (i.e., the Central Institute of Mental Health Mannheim and the university of Heidelberg) are factually inseparable from each other.
