# Supplementary material for: The research landscape of bipolar disorder in Germany: productive, but underfunded
Source: Int J Bipolar Disord. 2024 Jun 15;12:22. doi: 10.1186/s40345-024-00344-9 (PMC11180073; doi:10.1186/s40345-024-00344-9)
Supplement: Supplementary file 2 — Supplementary Material 2 [file 40345_2024_344_MOESM2_ESM.docx]

*Most cited articles*

Analysis of highly cited and thus “key” publications may give additional insights to main themes and topics within the field. Concerning our database, ten of the 20 most cited articles (50%) deal with psychiatric genetics, especially with findings from genome-wide association studies. This further supports a change in thematic trends in favor of psychiatric genetics. The remaining ten articles were mainly of methodological (e.g., rating scales, diagnostic manuals; N=6,3%) or epidemiological nature (N=2, 10%) as epidemiological information is frequently given in introductions and standardized measures were used for diagnostic purposes. See Table S1 for a list of the 20 publications that were cited most frequently.

*Table S1. The 20 most cited articles within the last decade of German research on bipolar disorder*

| Title | Topic |  | TC | DOI |
| --- | --- | --- | --- | --- |
| A rating scale for mania: reliability, validity, and sensitivity | Rating scale for mania | Young, 1978, Br J Psychiatry | 175 | 10.1192/bjp.133.5.429. |
| A rating scale for depression | Rating scale for depression | Hamilton, 1960, J Neurol Neurosur PS | 131 | 10.1136/JNNP.23.1.56 |
| Biological insights from 108 schizophrenia-associated genetic loci | GWAS study on genetic polymorphisms associated with SZ | Ripke, 2014, Nature | 129 | DOI 10.1038/NATURE13595 |
| The positive and negative syndrome scale (PANSS) for schizophrenia | Rating scale for SZ symptoms | Kay, 1987, Schizophrenia Bull | 104 | DOI 10.1093/SCHBUL/13.2.261 |
| Large-scale genome-wide association analysis of bipolar disorder identifies a new susceptibility locus near ODZ | GWAS study on genetic polymorphisms associated with BD | Sklar, 2011, Nat Genet | 100 | DOI 10.1038/NG.943 |
| Common polygenic variation contributes to risk of schizophrenia and bipolar disorder | Close genetic relationship between SZ and BD | Purcell, 2009, Nature | 99 | DOI 10.1038/NATURE08185 |
| Diagnostic and statistical manual of mental disorders: DSM-5™, 5th ed. | Diagnostic Manual | APA, 2013, Diagn Stat Man Ment | 98 | DOI 10.1176/APPI.BOOKS.9780890425596 |
| Identification of risk loci with shared effects on five major psychiatric disorders: a genome-wide analysis | Close genetic relationship between different psychiatric disorders | Smoller, 2013, Lancet | 90 | DOI 10.1016/S0140-6736(12)62129-1 |
| PLINK: a tool set for whole-genome association and population-based linkage analyses | Large data sets; GWAS studies; genome-wide associations | Purcell, 2007, Am J Hum Gen | 84 | DOI 10.1086/519795 |
| Genetic relationship between five psychiatric disorders estimated from genome-wide SNPs | Close genetic relationship between different psychiatric disorders | Lee, 2013, Nat Genet | 83 | DOI 10.1038/NG.2711 |
| Common genetic determinants of schizophrenia and bipolar disorder in Swedish families: a population-based study | Close genetic relationship between SZ and BD | Lichtenstein, 2009, Lancet | 77 | DOI 10.1016/S0140-6736(09)60072-6 |
| Collaborative genome-wide association analysis supports a role for ANK3 and CACNA1C in bipolar disorder | Psychiatric genetics; ion channelopathies involved in pathogenesis of BD | Ferreira, 2008, Nat Genet | 75 | DOI 10.1038/NG.209 |
| Reliability and validity of the Mini International Neuropsychiatric Interview for Children and Adolescents (MINI-KID) | Diagnostic Interview for Children and Adolescents | Sheehan, 1998, J Clin Psychiat | 72 | DOI 10.4088/JCP.09M05305WHI |
| Prevalence and Correlates of Bipolar Spectrum Disorder in the World Mental Health Survey Initiative | Epidemiology of BD | Merikanas, 2011, Arch Gen Psychiat | 68 | DOI 10.1001/ARCHGENPSYCHIATRY.2011.12 |
| Risk of metabolic syndrome and its components in people with schizophrenia and related psychotic disorders, bipolar disorder, and major depressive disorder: a systematic review and meta-analysis | Metabolic syndrome is similarly elevated in different psychiatric diagnoses and influenced by antipsychotic medication | Vancampfort, 2015, World Psychiatry | 60 | DOI 10.1002/WPS.20252 |
| A new depression scale designed to be sensitive to change | Depression rating scale that is particularly sensitive to treatment effects | Montgomery, 1979, Brit J Psychiat | 56 | DOI 10.1192/BJP.134.4.382 |
| The endophenotype concept in psychiatry: etymology and strategic intentions | Topic; genetic endophenotypes | Gottesman, 2003, Am J Psychiat | 55 | DOI 10.1176/APPI.AJP.160.4.636 |
| Genome-wide Association Study Identifies Genetic Variation in Neurocan as a Susceptibility Factor for Bipolar Disorder | Neurocan, an extracellular matrix glycoprotein as genetic polymorphism associated with BD | Cichon, 2011, Am J Hum Genet | 54 | DOI 10.1016/J.AJHG.2011.03.001 |
| Genome-wide association study identifies five new schizophrenia loci | Close genetic relationship between SZ and BD | Ripke, 2011, Nat Genet | 53 | DOI 10.1038/NG.940 |
| Lifetime and 12-month prevalence of bipolar spectrum disorder in the National Comorbidity Survey replication | Epidemiology of BD | Merikangas, 2007, Arch Gen Psychiat | 49 | DOI 10.1001/ARCHPSYC.64.5.543 |

*Note:* TC = Total number of citation.
